# Stochastic De-repression of Rhodopsins in Single Photoreceptors of the Fly Retina

**DOI:** 10.1371/journal.pcbi.1002357

**Published:** 2012-02-02

**Authors:** Pranidhi Sood, Robert J. Johnston, Edo Kussell

**Affiliations:** 1 Center for Genomics and Systems Biology, Department of Biology, New York University, New York, New York, United States of America; 2 Center for Developmental Genetics, Department of Biology, New York University, New York, New York, United States of America; 3 Department of Physics, New York University, New York, New York, United States of America; Princeton University, United States of America

## Abstract

The photoreceptors of the Drosophila compound eye are a classical model for studying cell fate specification. Photoreceptors (PRs) are organized in bundles of eight cells with two major types – inner PRs involved in color vision and outer PRs involved in motion detection. In wild type flies, most PRs express a single type of Rhodopsin (Rh): inner PRs express either Rh3, Rh4, Rh5 or Rh6 and outer PRs express Rh1. In outer PRs, the K_50_ homeodomain protein Dve is a key repressor that acts to ensure exclusive Rh expression. Loss of Dve results in de-repression of Rhodopsins in outer PRs, and leads to a wide distribution of expression levels. To quantify these effects, we introduce an automated image analysis method to measure Rhodopsin levels at the single cell level in 3D confocal stacks. Our sensitive methodology reveals cell-specific differences in Rhodopsin distributions among the outer PRs, observed over a developmental time course. We show that Rhodopsin distributions are consistent with a two-state model of gene expression, in which cells can be in either high or basal states of Rhodopsin production. Our model identifies a significant role of post-transcriptional regulation in establishing the two distinct states. The timescale for interconversion between basal and high states is shown to be on the order of days. Our results indicate that even in the absence of Dve, the Rhodopsin regulatory network can maintain highly stable states. We propose that the role of Dve in outer PRs is to buffer against rare fluctuations in this network.

## Author Summary

Complex networks of genetic interactions govern the development of multicellular organisms. One of the best-characterized networks governs the development of the fruit-fly retina, a highly organized, three-dimensional organ composed of a hexagonal grid of eight types of photoreceptor neurons. Each photoreceptor responds to a particular wavelength of light depending on the Rhodopsin protein it expresses. We present novel computational methods to quantify cell-specific Rhodopsin levels from confocal microscopy images. We apply these methods to study the effect of the loss of a key repressor that ensures each photoreceptor expresses only one Rhodopsin. We show that this perturbation has cell-specific effects. Our measurement of the cell-type specific Rhodopsin distributions reveals differences between photoreceptor cells, which could not otherwise be detected. Using mathematical models of gene expression, we attribute this variability to stochastic events that activate Rhodopsin production.

## Introduction

**Figure 1:**
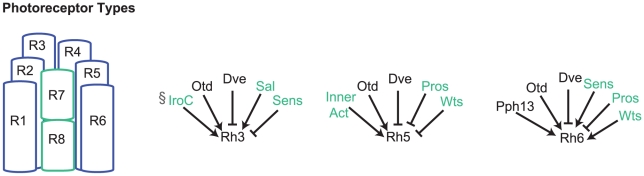
Schematic view of ommatidial organization and known regulators of Rhodopsins. (left) The eight photoreceptor (PR) types (R1–R8) are shown in their stereotypical arrangement within an ommatidium; outer PRs shown in blue, inner PRs shown in cyan. (right) Direct regulators of Rhodopsins Rh3, Rh5, and Rh6 are shown. Regulators that are expressed specifically in the inner PRs are shown in cyan. The symbol § indicates regulators known to exhibit spatial dependencies across the retina.

The ability of *Drosophila* to perceive color and motion depends on the specific patterning of several Rhodopsin proteins throughout its retina [1]–[3]. The fly retina is a complex three-dimensional structure that consists of a lattice of approximately 800 simple eyes known as ommatidia [4]. As shown in Figure 1, each ommatidium is a bundle of eight photoreceptor neurons (PRs), with six motion detecting PRs (R1–R6) on the perimeter (“outer” PRs) and two smaller, color detecting PRs (R7 & R8) in the middle (“inner” PRs) [5]–[12]. Beginning at the third instar larva, photoreceptors arise following the passage of a morphogenetic furrow across the eye imaginal disc, a monolayer of epithelial cells. As the furrow passes, cells are recruited to ommatidia in a stereotyped manner wherein the R8 photoreceptor is recruited first and then followed by pairs of outer photoreceptors: R2 and R5, R3 and R4, then R1 and R6. Finally, R7 joins the group of cells. During pupation, photoreceptors express specific Rhodopsins (for details of the process, see [13], [14]).

A well-studied genetic network controls Rhodopsin protein expression in the eight PR cell types, and enforces a “one-neuron, one-receptor” rule across the majority of the retina, such that each PR expresses one and only one of five types of Rhodopsin proteins (Rh1, Rh3, Rh4, Rh5, or Rh6) [15]. In outer PRs, each cell expresses Rh1 exclusively. There are two major types of ommatidia: in a random subset consisting of approximately 35% of ommatidia, inner PRs exhibit coupling such that when the R7 cell expresses Rh3, the R8 cell expresses Rh5; in the other 65% of ommatidia, when R7 expresses Rh4, R8 expresses Rh6 (for exception, see [16]).

Several regulators of Rhodopsin patterning have been discovered and their regulatory interactions are well-characterized [16]–[21]. The K_50_ homeodomain protein Defective proventriculus (Dve) was recently shown to enforce the “one-neuron, one-receptor” rule in the outer PRs and in the subset of Rh4-expressing R7 cells [22]. In outer PRs, Dve acts together with the activator Orthodenticle (Otd) in an incoherent feedforward loop motif to repress Rh3, Rh5, and Rh6. In the inner PRs, a second coherent feedforward loop that includes the inner PR factor Spalt (Sal), represses Dve thus allowing Rhodopsin expression. In *dve* mutants, Rh3, Rh5 and Rh6 are de-repressed in outer PRs at levels that vary among cells. Importantly, at the time of Rh expression, Dve is expressed in outer PR cell types where it represses Rh3, Rh5 and Rh6 (Figure 1). Dve's effect on Rhodopsin expression, however, is modulated by cell-type specific inputs onto the promoters of each *rhodopsin* gene (Figure 1 and [22]). Most of these inputs have previously been shown to affect *rhodopsin* expression in inner photoreceptors only. However, Otd and Hazy/Pph13 (a Q_50_ homeodomain protein) are expressed in all PRs similarly to Dve [18], [21], [23]. Both Otd and Hazy/Pph13 have been shown to be necessary but not sufficient factors for expression of specific *rhodopsins in vivo* (Figure 1) and sufficient to activate their expression *in vitro* [24].

**Figure 2:**
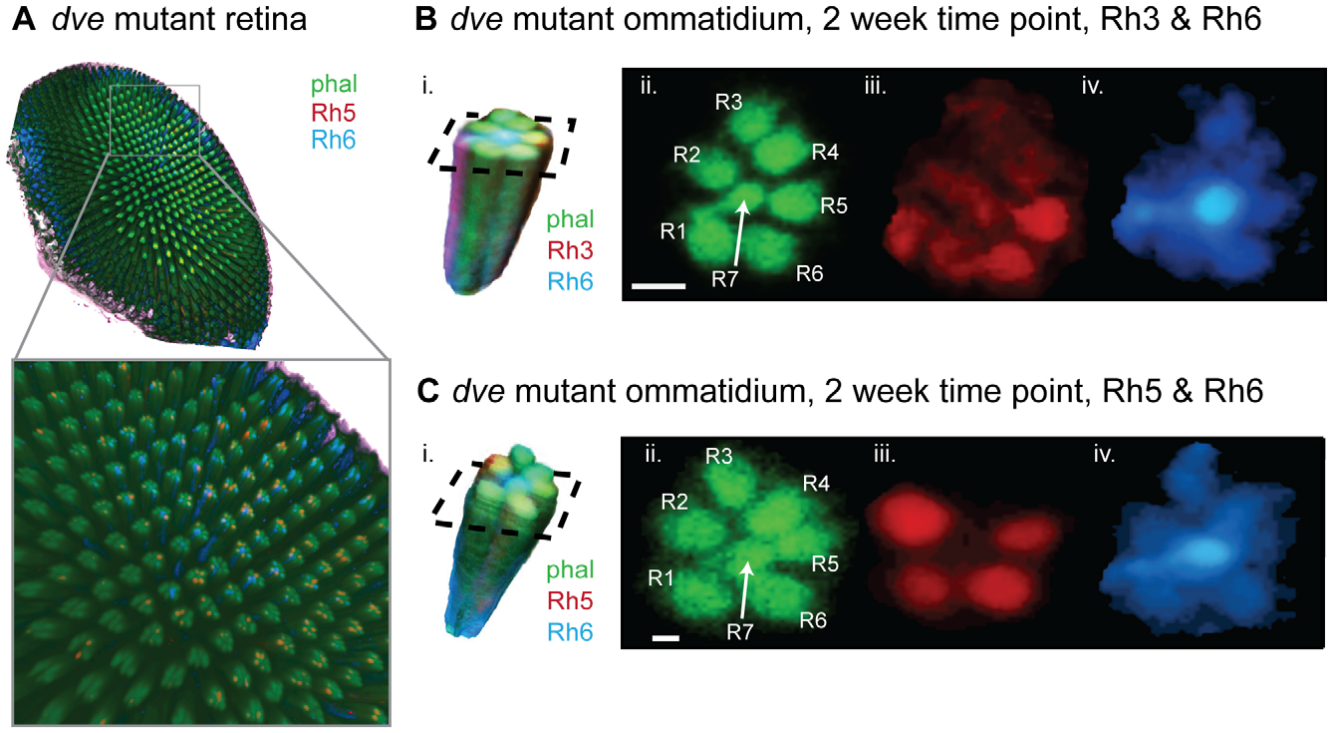
Rh3, Rh5 and Rh6 are de-repressed in *dve* mutants. (A) Three dimensional rendering of a representative confocal stack of a retina dissected at the 2 week developmental time point. Phalloidin, which stains actin, was used to visualize the rhabdomeres (green). This retina is co-stained for Rh5 (red) and Rh6 (blue). The inset shown is a zoomed-in view of the center of the retina. (B & C) Retinae were co-stained for two Rhodopsins, and representative ommatidia extracted automatically from the image stacks are shown for retinae co-stained for either Rh3–Rh6 or Rh5–Rh6 (panels B-i & C-i). Panels B-ii & C-ii show a cross section of the ommatidium in the phalloidin channel, indicating the automatically identified PR cells (R1–R6; R7/R8). Rh3 levels exhibit de-repression in outer PR cells (B-iii). Rh6 levels exhibit de-repression in outer PR cells (panels B-iv & C-iv). Rh5 levels exhibit de-repression in outer PR cells (C-iii). Scale bar is 1.5 µm (panels B) and 1.0 µm (panels C).

Here, we quantitatively studied a molecular null mutation in *dve*. Since the variable nature of this phenotype requires a quantitative analysis (Figure 2), we developed image analysis algorithms to identify each ommatidium in the retina and discriminate individual PRs in 3D confocal stacks of retinae. We applied these methods to quantify cell-specific effects in *dve* mutants in thousands of cells by measuring relative protein levels for the three Rhodopsins in the eight different PR types over a time course of four weeks. We measured a wide cell-to-cell variability in Rhodopsin expression. Our ability to precisely quantify Rhodopsin levels enables detection of subtle differences among the outer PR cell types, which manifest in the de-repression of Rhodopsins. We use stochastic models to understand the underlying causes of the observed Rhodopsin distributions. This allows us to attribute differences among cells to the rates of molecular processes such as protein and mRNA synthesis and degradation rates. On the basis of our modeling, we propose a functional role for Dve in outer PRs as a buffer against rare fluctuations in the Rhodopsin regulatory network.

## Results

### Image Analysis, Quantification, and Reproducibility across Replicates

**Figure 3:**
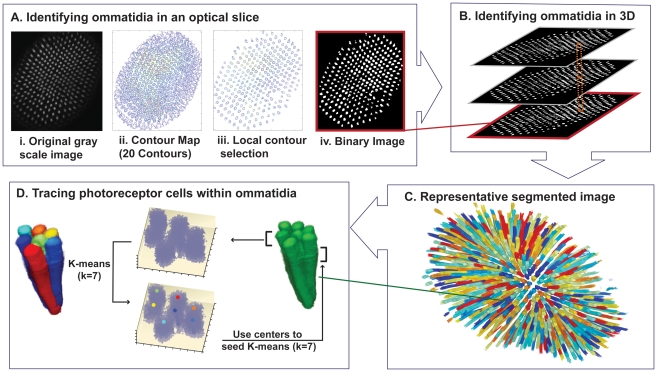
Image analysis methods to identify ommatidia and individual PRs. (A) Step 1 – *Identifying ommatidia in an optical slice*. Each optical section is thresholded using local contour selection. i. a representative gray scale optical section; ii. a contour map of this image, where 20 intensity contour levels are drawn; iii. results of contour selection around a region; iv. the resulting binary image. (B) Step 2 – *Identifying ommatidia in 3D*. Filament finding in three dimensions is applied by searching in five consecutive slices for overlapping pixels, and growing the overlapping regions in subsequent slices. (C) Representative segmented image that results from steps 1 and 2. (D) Step 3 – *Tracing PR cells within ommatidia*. A representative ommatidium, extracted from the retina. Choosing the top 800 most intense points in the first five slices, k-means clustering is performed to identify 7 clusters, corresponding to the 7 PRs in each slice. Using these centers as seeds, we shift the window of five slices by one slice and perform k-means of the top 800 most intense points. This process is continued until the PRs are traced. Representative results are depicted on the left of the panel where each color identifies a different cell.

**Figure 4:**
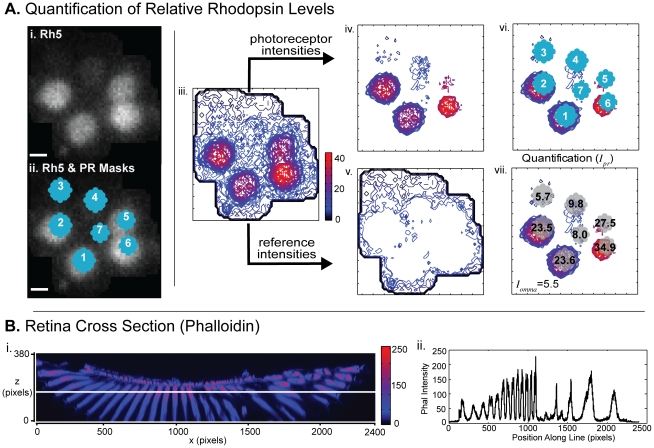
Quantification of relative Rhodopsin levels. (A) i. an optical section of an ommatidium stained for Rh5; ii. the optical section with PR masks whose centers are identified by the clustering method described here; iii. contour map of Rh5. Intensities are colored such that low intensities are dark blue and high intensities are bright red. To calculate intensity measures, our method only uses contour lines that overlap with PRs. The contour map is broken into contours that localize to PRs (panel iv) and those that constitute the background levels (panel v). vi. shows the overlay of the contours that localize to PRs together with the masks; vii. the calculated intensity measures are shown for each PR in this slice. Final intensity measurements are averaged over an automatically identified z-range. (B) i. A cross section of the retina in the xz-plane in the phalloidin channel showing the effect of tissue depth on intensity measurements. Intensity level is indicated by color from highest (red) to lowest (blue); ii. A plot of the intensities along the line shown in panel i. The brightest points in the xy plane are close to the center of the retina where light has less tissue to travel through.

Thirty *dve* mutant retinae collected at four different developmental time points were analyzed by confocal microscopy and automated image analysis algorithms (see Methods). We developed algorithms that automatically analyze three-dimensional confocal stacks of entire retinae and computationally extract ommatidia from the stacks (Figure 3). Within each ommatidium, the algorithms identified photoreceptor cells and assigned the correct photoreceptor type. For most retinae, our algorithms identified >500 ommatidia, and the number that were sufficiently well-resolved to allow automatic identification of individual photoreceptors was typically in the range 70–200 ommatidia per retina. We quantified Rhodopsin protein levels using a local relative intensity measure (*I_l_*) across an interval of z slices identified by the algorithm to maximize both the number of ommatidia with well-resolved PRs and the number of slices used for quantification (Figure S1 and Text S1). This intensity measure, *I_l_*, is calculated in each z slice such that each PR's intensity value (*I_pr_*) is normalized by the local ommatidia background (*I_omma_*), and averaged over the z slices: *I_l_* = <*I_pr_*/*I_omma_*>. In order to quantitatively compare Rhodopsin protein levels across individual retinae, we overcame several technical challenges resulting from imaging within the complex retinal tissue (see Methods and Figure 4). Using *I_l_* to quantify protein levels, we demonstrated that Rhodopsin distributions are reproducible across replicates (see Methods and Figure S2).

### Cell-Specific Expression Dynamics

We applied our quantification approach using *I_l_* to measure cell-specific Rhodopsin expression in *dve* mutants across several time points. By comparing the measured distributions for different Rhodopsins in different PRs, we observed PR specific effects in de-repression.

#### Inner photoreceptors (R7, R8)

The wild-type expression pattern in R7 cells is bimodal such that approximately 35% of cells exclusively express Rh3, while the other 65% exclusively express Rh4. Similarly, a 35∶65 ratio of Rh5∶Rh6 is observed in R8 cells, due to a signal from R7 to R8. We previously showed that in the *dve* mutant the bimodal expression pattern of Rh4 is unaffected, but the Rh3 pattern is eliminated, with Rh3 expressed at a high level in all R7 cells [22]. In R8 cells, however, the bimodal expression of both Rh5 and Rh6 remains. The R8 cells in the *dve* mutant therefore provide a useful baseline for quantification of Rhodopsin expression; since Rh6 is expressed at high levels in one subset of R8 cells and is repressed in the remaining R8s, *I_l_* should reflect this bimodality. Furthermore, this distribution can be used to determine the range of *I_l_* values that correspond to the ‘on’ state of Rhodopsin expression.

**Figure 5:**
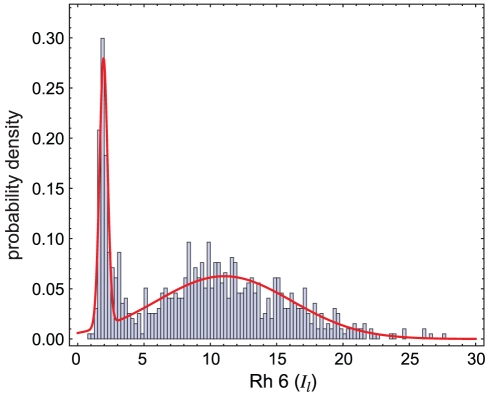
Bimodal expression of Rh6 in R8 cells. The distribution of Rh6 levels in R8 cells shown here represents retinae at all time points. The bar graph indicates the probability density of all PRs in each expression level bin. A bimodal distribution, given by a mixture of two normal distributions, was fit to the data using maximum likelihood fitting. The means (μ) and standard deviations (σ) of the two Gaussians are shown, and the mixture proportion is such that 80% of cells express Rh6 at high levels, while 20% express Rh6 at low levels.

In Figure 5 we show the distribution of Rh6 in R8 cells measured in a representative sample of retinae across all time points. This distribution exhibits two pronounced peaks. We fit a mixture of two normal distributions to the pooled data, and found that 80% of cells express Rh6 at high levels. The ‘off’ state of Rh6 has a basal level of *I_l_*∼1.9±0.3 (mean±stddev), while the on state has *I_l_*∼11.0±5.1, and we had enough data to observe cells at 3 standard deviations above the mean, at *I_l_*>26. The data fit well to the mixture distribution, with a Kolmogorov-Smirnov test statistic of 0.09 (p-value = 0.42). Holding constant the inferred parameters of the two normal distributions, we refit only the mixture parameter (the proportion of cells in the on state) separately for each retina. This yielded a mean proportion of 78% on, with a standard deviation of 3.5% across retinae. The approach we developed here to characterize variability of the ratio for *dve* will be useful in future studies of the wild-type ratio.

Our quantification in inner PRs here determined the range of intensity values corresponding to the on and off states of Rhodopsin expression. On the basis of previous measurements of Rhodopsin density in rhabdomeres, we estimated the conversion from our intensity units into numbers of Rhodopsin molecules [25]. Rhodopsin density in the rhabdomere was measured to be 2.5×10^5^ molecules/µm^3^. Using the average *I_l_* for the on state, we find that one intensity unit corresponds to approximately 2.3×10^4^ molecules/µm^3^.

#### Outer photoreceptors (R1–R6)

**Figure 6:**
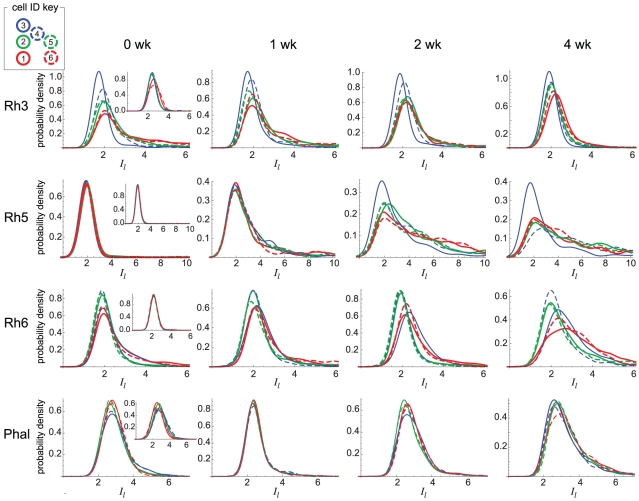
Cell-specific expression dynamics of Rhodopsins. Rhodopsin level distributions are shown for each PR type and each Rhodopsin, pooled over retinae at each time point. Each curve corresponds to a different outer PR (R1–R6), with colors and dashing indicated by the cell ID key. Histograms were smoothed for ease of comparison. A Gaussian smoothing kernel, 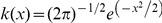, is used to construct the smoothed density *P*(*x*):
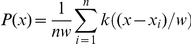
where *x_i_* denote the observed data points, *n* is the number of data points (see Table 1), and *w* is the smoothing bandwidth (*w* = 0.3 for Rh3, Rh6, and Phalloidin; 0.5 for Rh5). Insets show distributions measured from two wild-type retinae. Phalloidin levels are shown in the bottom row. Number of retina in each panel (0 week, 1 week, 2 week, 4 week) is as follows: Rh3: (4, 2, 3, 3); Rh5: (3, 2, 7, 3); Rh6: (7, 4, 10, 6).

Wild-type outer PRs express Rh1 exclusively and strongly repress Rh3, Rh5, and Rh6. In *dve* mutants, these three Rhodopsins are de-repressed in outer PRs (Figure 2). While clear functional differences exist between the outer (motion-detecting) and inner (color-detecting) PRs, differences among outer PRs have not been established with regards to Rhodopsin regulation. Our quantitative approach could detect subtle differences in Rhodopsin expression among outer PRs. Figure 6 shows the cell-specific Rhodopsin expression distributions we measured at different time points (smoothed histograms are shown). Comparison with Figure 5 indicates that in all PRs, across Rhodopsins and time points, high expression levels such as those observed for Rh6 in R8 are rarely seen in outer PRs. In the next section, we show that infrequent stochastic activation events can account for the observed expression level distributions.

The Rhodopsin distributions reveal significant differences among outer PRs (Figure 6). While mean Rhodopsin levels are similar across PRs, the differences are apparent in the distribution shapes, specifically the location of their mode and the width of their tails. To test whether visually apparent differences have statistical significance, we used a one-sided, two-sample Kolmogorov-Smirnov test, a non-parametric test for differences in the shapes of distributions. We tested for differences between each PR and all other PRs. We also tested for differences between multi-PR subsets. Using this test, we found several striking cell-specific Rhodopsin expression patterns (see Table S1 for p-values):

1. Rh3 tends to be more repressed in R3 cells than in all other PRs at all four time points. Similarly, Rh3 tends to be more repressed in R4 cells than in all other PRs until the fourth time point.
2. Rh5 tends to be more repressed in R3 cells than in all other PRs, most significantly at the 2 and 4 week time points. For Rh5, R3 cells are the only cells that remain significantly repressed at 2 and 4 weeks; all other cell types tend to express Rh5 at high levels.
3. Rh6 expression in R2, R4 and R5 cells are significantly more repressed than a group composed of R1, R6 and R3 at all time points.
4. For Rh3, R1 and R6 cells tend to be less repressed at all time points.
5. Rh6 expression in R1 cells exhibits strong de-repression at all time points.

We hypothesized that some of these cell-specific differences might result from similar levels of regulators when cells are developmentally recruited. To test this, we compared the distributions of co-recruited pairs (each shown in a different color in Figure 6), and found that these pairs tend to behave more similarly to each other than to the other PRs. The effect is most visually striking among Rh3 distributions. Using a one-side Kolmogorov-Smirnov test (see Table S1 for p-values), we find that the R3 and R4 cells tend to repress Rh3 more strongly than the R2 and R5 pair of cells. We also find that the R2 and R5 pair tend to repress Rh3 more strongly than the R1 and R6 pair. Co-recruited pairs of photoreceptors do not exhibit similar tendencies in their distributions of Rh5 and Rh6. To test if the tendency we observe in Rh3 expression can be explained by differences in cellular structure, we compared cell-specific distributions of Phalloidin, which binds actin filaments. We observe a slight tendency for pairs of co-recruited cells to have similar distributions, however this tendency is not as significant either visually or statistically as that observed for Rh3 (see Table S1).

### Stochastic Modeling of Relative Rhodopsin Levels

**Figure 7:**
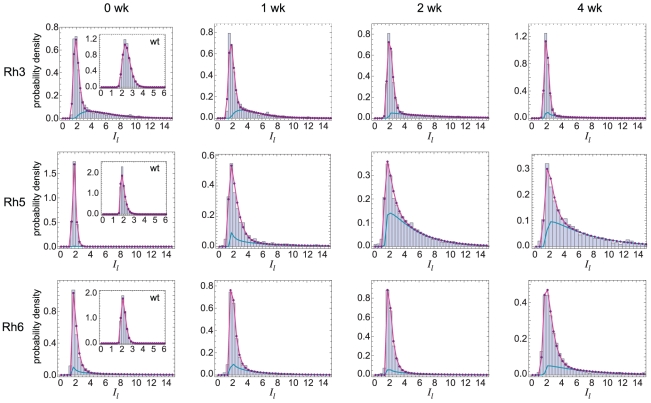
Rhodopsin distributions in photoreceptors fit to a two-state gene expression model. Data pooled across photoreceptors is shown in bars. The fit to the two-state model, 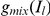, given in Table 1, is shown in magenta; the component corresponding to the on state, 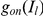, is shown in cyan. Densities 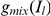 and 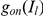 are integrated over each bin before plotting to allow comparison with the bar histograms. Insets show data from wild-type retinae, and the fit to a single gamma distribution function (Table 1) is shown in magenta.

The distribution of Rhodopsin protein levels we measured should be informative of the processes of mRNA and protein production and degradation for different *rhodopsin* genes. Previous studies have derived the form of the equilibrium distributions for several different models [26]–[28]. In the simplest model, a constitutively active promoter produces transcripts that are translated into protein [27]. Assuming that protein lifetimes are significantly longer than mRNA lifetimes, the equilibrium distribution of protein levels is a gamma distribution, 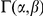, with shape parameter *α* and scale parameter *β*. The two parameters are related to the rates of mRNA production (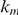) and degradation (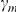), and of protein production (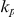) and degradation (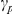) as follows: 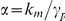, 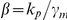. We expect this model to be appropriate when transcriptional activity is uniform across cells. For example, since wild-type flies strongly repress Rh3, Rh5, and Rh6 in outer PRs, their very low expression levels in those cells should be well-modeled by a uniform basal transcription rate. To test this, we measured Rhodopsin distributions in wild-type flies. We found excellent fits to gamma distributions for all three Rhodopsins, shown in Figure 7 (insets) and Table 1. The difference between Rh3 and Rh5 was pronounced in their values of *α* (they had identical values of *β*), indicating differences in their basal promoter activities or protein turnover rates, rather than differences in translation or RNA stability.

**Table 1:** Best-fit parameters for two-state model of gene expression.

	Rh3				Rh5				Rh6			
*dve*	0 wk	1 wk	2 wk	4 wk	0 wk	1 wk	2 wk	4 wk	0 wk	1 wk	2 wk	4 wk
*α_off_*	5.6	5.6	5.6	9.0	2.0	1.8	1.7	1.7	1.2	1.4	1.3	1.9
*β_off_*	0.17	0.17	0.17	0.10	0.17	0.60	0.58	0.59	0.40	0.41	0.44	0.55
*α_on_*	2.0	1.9	1.0	0.59	0.32	0.63	1.1	1.1	0.53	0.77	0.68	1.1
*β_on_*	2.1	1.8	5.8	5.3	41	10	3.3	4.8	11	4.3	5.9	5.0
*p_on_*	0.34	0.35	0.32	0.20	0.02	0.31	0.68	0.65	0.25	0.30	0.19	0.32
Δ*x*	1.09	1.05	1.24	1.20	1.62	1.20	1.20	1.20	1.65	1.65	1.65	1.40
*n*	2,737	1,169	1,736	2,163	1,687	950	2,616	1,673	4,422	2,121	4,353	3,836
***wt***	**0 wk**				**0 wk**				**0 wk**			
*α*	5.6				2.2				3.9			
*β*	0.17				0.17				0.12			
Δ*x*	1.57				1.62				1.72			
*n*	672				609				672			

All fits were performed using maximum likelihood optimization (we used *Mathematica 8* to perform the fitting). The functional form of the fit consisted of a linear combination of two gamma distribution pdfs, denoted 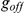 and 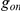, with the mixture parameter 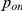. The pdf for a gamma distribution is defined by 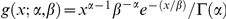. The 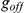 pdf is shifted by Δ*x*, determined by the lowest levels detected in each experiment: 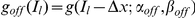. The 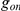 pdf is shifted to the mode of the 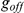 pdf: 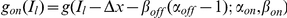. The mixture pdf is given by 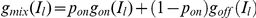.

In contrast to these results from wild-type retinae, the distributions in *dve* retinae were not fit as well by this simple model (see Figure S3). While in some cases the fit was good at the tails of the distributions, the fit at the modes exhibited significant deviations. The simple model, therefore, predicts fewer cells expressing low levels of Rhodopsin than observed. Comparing *dve* and wild-type distributions in Figure 7, appears that the mode corresponds to the subpopulation of cells that exhibit basal expression.

These deviations from the simple model indicate that protein expression is strongly non-uniform across cells. One possibility is that Rhodopsin promoters can interconvert reversibly between on and off states, and mRNA is produced only when the promoter is on. In several different regimes of this promoter on/off interconversion model, protein levels are predicted to exhibit a gamma distribution. For example, if the gene's inactivation rate is much larger than both the activation rate and the mRNA degradation rate, then mRNA levels will have a gamma distribution; hence, if protein levels closely track mRNA, the gamma distribution will be observed [26]. Likewise, if the promoter state interconverts significantly faster than proteins are degraded, a gamma distribution is predicted [28]. Our data exhibits significant deviations from both gamma distributions (Figure S3) as well as the general solution obtained in [28] (data not shown).

Our data are much more consistent with a two-state network model in which proteins can be produced at either a low, basal rate (off state) or a high rate (on state), and interconversion between the two states is much slower than all other processes. We emphasize that these two states are not necessarily different states of the promoter alone – they may result from differences in other processes and thus correspond to the overall state of the network that regulates Rhodopsin production. Due to the slow interconversion between states, proteins levels in each state exhibit a gamma distribution; the overall distribution is a mixture of two gamma distributions, with parameter *p_on_* denoting the fraction of cells in the on state.

Fits to the two-state model are shown in Figure 7, where the blue line indicates the distribution for cells in the on state (parameter values are given in Table 1). While such mixture models can suffer from over-fitting, here we have avoided this problem by explicitly measuring the wild-type distributions, which correspond to the off state (Figure 7, inset). The wild-type off state is similar in its position and shape to the *dve* off state across time points, indicating that the fits are reliable.

The model allows us to infer the fraction of cells in the on state at each time point (Table 1). For Rh5, we see initially a very small fraction of cells are on (*p_on_* = 0.02); over the course of 2 weeks, cells get induced until approximately 2/3 of cells are on, a value that is maintained through the 4 week time point. For Rh3, *p_on_* remains at ∼0.3 until 4 weeks, when it decreases to 0.2. For Rh6, *p_on_* fluctuates in the range 0.2–0.3 over the time course. The difference between the on and off states of cells, and further implications of our modeling, are addressed in detail in Discussion.

### Pairwise Rhodopsin Correlations

The existence of common regulatory mechanisms acting on different genes can often be inferred by measuring correlations of their expression levels within cells. Although Otd regulates three different *rhodopsin* genes, the presence of Dve in wild-type retinae maximally represses these genes in outer PRs, and correlations cannot be observed. In *dve* mutants, Rhodopsin expression is revealed in outer PRs. If Otd were acting alone, we would expect that fluctuations in Otd levels would lead to positive correlations between different pairs of Rhodopsins. However, the presence of other regulators in different outer PR cells could modulate the strength or even the sign of correlations.

**Figure 8:**
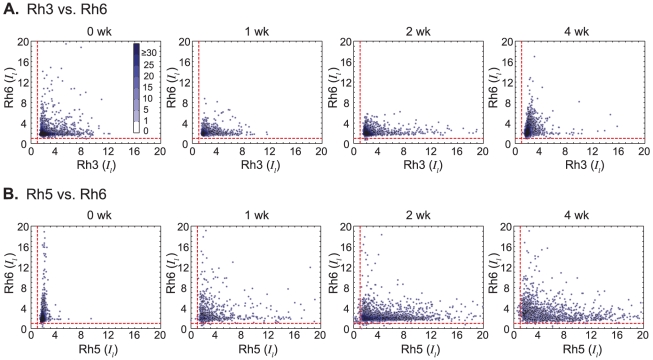
Distribution of pairwise Rhodopsin expression. (A & B) Each point corresponds to a single PR, with the two coordinates giving the relative expression levels of two Rhodopsins. Data was pooled across all replicates at each time point. To give a sense for the density of points in different regions, each point was colored to indicate the number of points within a radius = 0.5 around it. The color bar shows the number of points indicated by each color.

To test this, we plotted the distribution of relative levels for pairs of Rhodopsins that were co-stained, Rh3–Rh6 and Rh5–Rh6 (Figure 8). In the pooled data, across all replicates and photoreceptors, we found statistically significant positive correlations for Rh3–Rh6 at the last time point (Spearman's ρ≈0.3, p-value<10^−4^) and negative correlations for Rh5–Rh6 (Spearman's ρ≈−0.35, p-value<10^−4^) (Figure S4, upper panels). To verify these, we examined correlations within each replicate and within each photoreceptor (Figure S4, lower panels). For Rh3–Rh6, although several replicates exhibit statistically significant correlations, there is no consistent pattern, although there is a strong tendency for positive correlations at the fourth week time point. Some positive correlations may be spurious, e.g. while the measure *I_l_* normalizes for variations in local mean intensity across the retina, higher-order intensity variations might account for weak positive correlation between Rh3–Rh6.

For Rh5–Rh6, on the other hand, we find reproducible anti-correlation across all three replicates at the 4 week time point, which are consistent across PR types. One explanation for anti-correlation could be exclusion of different Rhodopsins due to limited space and dense packing within the rhabdomere. In that case, we would expect a cloud of points slightly elongated along a line of negative slope. Figure 8 is not consistent with this scenario. Instead, along the increasing Rh5 axis in the 4 week panel, we see a spread of points, with decreasing density in the Rh6 direction. Cells that highly express one Rhodopsin, tend to express the other type at a lower level, and there are few cells that co-express both Rhodopsins at high levels. We conclude the Rh5–Rh6 anti-correlation is the result of factors other than Otd acting in the outer photoreceptors (see Discussion).

## Discussion

In this paper, we established the *Drosophila* compound eye as a quantitative system for studying cell-specific gene expression. We developed methods to image the complex three-dimensional tissue and automatically identify ommatidia and their constituent PRs. We quantified the cell-specific effects of removal of *dve*, a key transcriptional repressor that regulates Rhodopsin patterning. In wild type outer PRs, which exclusively express Rh1, Dve represses the expression of Rh3, Rh5, and Rh6.

Our data shows that removal of Dve leads to a continuous and wide distribution of expression levels (Figure 7). This cell-to-cell variability exhibits PR-specific differences (Figure 6) suggesting that each cell type may express different levels of Rhodopsin regulators (Figure 1).

Importantly, we could only detect these differences by comparing distributions since mean Rhodopsin levels exhibit little change among outer PRs. The fact that Rh3 repression is greatest in R3 and R4 cells is consistent with the fact that the R3/R4 pair is known to undergo additional differentiation after PRs are recruited [29]. Thus, our analysis has revealed that cell fate differences in R3 and R4 yield distinct Rh3 expression in *dve* mutants. Furthermore, other co-recruited pairs (R1/R6 and R2/R5) also appear to regulate Rh3 levels similarly, suggesting that each pair may have similar levels of Rh3 regulators. Surprisingly, however, such similarities between co-recruited pairs are not detected for Rh5 and Rh6. This observation suggests that outer PRs exhibit differences in the amount and types of Rhodopsin regulators they contain. For example, the R3 cell exhibits a strong tendency to repress Rh5, which is distinct from all other PRs (Figure 6). These differences in Rhodopsin regulation in outer PRs have not previously been shown. While some examples of differential gene expression among outer PR types are known [30]–[32], further study of Rhodopsin regulation in outer PRs is needed to identify the molecular basis for the differences we observe.

We showed that Rhodopsin distributions in outer PRs are consistent with a two-state model in which Rhodopsin production occurs at either high or basal rates, which we call respectively the on and off states (Figure 7). To understand the biological basis for these two states, it is instructive to compare the model parameters *α* and *β* between them, across time points, for each Rhodopsin (Table 1). In all cases, we find 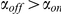 and 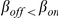. The most pronounced differences are between *β* values, which are an order of magnitude or more larger in the on state than the off state at most time points. Since 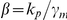 is the ratio of protein translation to mRNA degradation rates, the model suggests that differences between the two states of cells in the *dve* mutant may stem from post-transcriptional regulation. The on state could be associated with stabilization of the transcript (reduction of 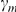) and/or increased translation (increase of 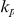). Differences in Rhodopsin trafficking to the rhabdomere may comprise additional post-transcriptional differences between the states. Because our experiments measure protein level distributions, they do not provide information about the transcription rate 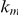 independently of 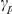, i.e. only the ratio 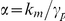 can be determined. Thus, transcriptional differences between on and off states cannot be inferred. However, under the reasonable assumption that 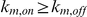, the observation that 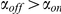 implies that Rhodopsin turnover rates (discussed below) increase significantly in the on state.

Our model assumes that on ↔ off switches occur infrequently compared to the equilibration timescale of the gene expression and protein translation dynamics. This timescale was shown to be of order 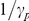 [28], and can be estimated from measured values of Rhodopsin turnover rates. The best measurements are of Rh1 in the blowfly, *Calliphora*, a dipteran with the same compound eye organization and patterning of *Drosophila*. The half-life of Rh1 depends on exposure to light: photo-activated Rh1 has a half-life of 2 hours, while in the dark its half life is 5 days [33]. Subsequent studies suggest that half-lives are longer in *Drosophila*, e.g. photo-activated Rh1 half-life is ∼13 hours [25]. Our flies were raised in 12 h light-dark cycles, hence 1 day is an approximate upper bound for the equilibration timescale. The rate constant for on ↔ off switching is therefore predicted to be significantly slower than 1 event per day per cell, a result that is fully consistent with our observation that changes in *p_on_* occur over the timescale of weeks (Table 1).

The timescale for on ↔ off switches, which is comparable to the organismal lifespan, is strikingly slow in view of other systems where stochastic activation occurs on the order of minutes [34], [35]. To some extent, this difference could result from the fact that photoreceptors are post-mitotic cells with overall slower metabolic processes than the actively dividing cells used in previous studies. More important in our view, however, is the fact that the default state of *rh3*, *rh5*, and *rh6* genes in outer PRs is off, and the strong Rhodopsin-specific activators are not expressed in these cells (Figure 1). Thus, the very slow timescale we infer for on ↔ off interconversion suggests that even in the absence of Dve, the Rhodopsin regulatory network in outer PRs can maintain two extremely stable states of Rhodopsin expression. We therefore propose that the functional role of Dve in these cells is to buffer against rare fluctuations in the Rhodopsin regulatory network.

While the mechanism by which Dve buffers against fluctuations is not known, we provide a simple toy network model in which buffering operates directly through Dve's known behavior as transcriptional repressor of *rhodopsin* genes. The mathematical model presented in Text S1 is constructed by analogy with well-known bistable networks such as the *lac* operon [36], [37]. The capacity of photoreceptors to produce large amounts of Rhodopsin protein requires up-regulation of the protein production machinery, which could be induced by Rhodopsin protein itself within a positive-feedback loop. In this case, once Rhodopsin levels increase beyond some threshold, protein production would kick into “high gear”, with concomitant increase in degradation pathways to allow for efficient turnover.

Depending on the Rhodopsin mRNA level, the system can be either bistable or monostable, as we show in Text S1. If Rhodopsin mRNA concentration is very low (or very high), the system has a single stable fixed point, corresponding to low (or high) production. In an intermediate range of mRNA levels, the system exhibits bistability (see Figure S5). In the bistable regime, a cell that is in the low production state remains stably in that state. An increase of Rhodopsin levels by rare fluctuation is required to drive the system into the high production state. Once there, it remains stably in high gear. Within this network, the role of Dve is to buffer against fluctuations [38] by ensuring that the system remains in the monostable regime. Removal of Dve increases mRNA levels, moving the system into the bistable range. Thus, in this model Dve exhibits the quintessential hallmark of a buffer: it controls the stability of the system, not its state (see Text S1).

Our toy network provides a plausible mechanism of buffering by Dve, but other scenarios are clearly possible, e.g. via additional regulatory interactions. The key point indicated by our results is that Rhodopsin production is not entirely determined by transcript levels. Removal of Dve renders the system poised for activation, making it susceptible to fluctuations. While such fluctuations can in principle occur without activators, it is known that the specific Rhodopsin activators Otd and Hazy/Pph13 are expressed in outer PRs [18], [21], [23]. The levels of these regulators and any others would thus be major determinants of the rate of fluctuations that drive the system from one stable state to the other.

Using our approach, we measured correlations between levels of different Rhodopsin proteins. While we observed weak but significant positive and negative correlations between both Rh pairings (Figure S4), only the negative correlations of Rh5 and Rh6 at the 4 week time point were consistent across replicates and cell types. It is noteworthy that in wild-type flies in the inner R8 cell, Rh5 and Rh6 expression is strongly bimodal due to the presence of a double negative feedback loop between *warts* and *melted* [19]. Transcriptional reporters of *warts* [19] and *melted* (D. Jukam, personal communication) are expressed in low levels in subsets of outer PRs, suggesting that the major effectors of this negative feedback loop are present in outer PRs. The presence of these regulators could result in the anti-correlations of Rh5 and Rh6 revealed in *dve* mutants. Moreover, recent work has shown that in R8 photoreceptors, Rh6 acting through an uncharacterized pathway has the capacity to inhibit Rh5 expression [39]. Removal of Rh6 leads to progressive expression of Rh5 in R8 PRs, which becomes apparent only after 2 weeks. Our finding of Rh5–Rh6 anti-correlation in *dve* outer PRs, which develops only after 2 weeks, suggests that a similar Rh6-mediated repression could be active in the outer PRs and revealed in the *dve* mutant.

Our results demonstrate the power of applying quantitative approaches to the study of systems-level problems in developmental biology. Our measurement of cell-specific Rhodopsin distributions enables detection of subtle differences among outer PR types, which were previously unknown. Our modeling of the distributions reveals that post-transcriptional processes play a major role in stochastic de-repression of Rhodopsins in outer PRs in the absence of Dve. More generally, we infer that the cellular state corresponding to basal Rhodopsin production is stably maintained by the Rhodopsin regulatory network even without Dve. On the basis of these findings, we conclude that Dve's role in outer PRs may be to act as a buffer against fluctuations in the genetic network that controls Rhodopsins.

## Methods

### *Drosophila* Retinae, Staging, and Sample Preparation

All retinae were dissected from *dve^186^* flies, a molecular null deficiency [22]. Flies were raised on standard corn meal-molasses-agar medium and grown at 25°C. Flies were staged and then dissected at 4 time points after eclosion: 0 weeks (+/−1 day), 1 week (+/−1 day), 2 weeks (+/−1 days), 4 weeks (+/−1 days). All retinae were stained with Alexa-488 conjugated phalloidin, which binds actin and is used to visualize the actin-dense rhabdomeres. Additionally, two antibodies are used to simultaneously visualize Rhodopsins: retinae were co-stained with one of two pairs, either mouse anti-Rh3 (1∶10) and rabbit anti-Rh6 (1∶2000), or mouse anti-Rh5 (1∶200) and rabbit anti-Rh6 (1∶2000). The fluorophores conjugated to secondary antibodies were Alexa 568 (Rh3), Alexa 568 (Rh5), and Alexa 633 (Rh6). Retinae were dissected and fixed for 15 minutes with 4% formaldehyde at 25°C. Retinae were rinsed twice, washed for at least 2 hours in PBX and then incubated overnight with the primary antibodies diluted in PBX. Retinae were then rinsed twice, washed in PBX for more than 4 hours and incubated overnight with secondary antibodies. Retinae were mounted in Prolong Gold following two additional rinses and a 2+ hour wash. All retinae were cured for at least 5 days but no more than 7 days. Prolong Gold exhibits the best refractive index matching between mounting media and objective oil.

### Microscopy and Image Acquisition

Images were acquired using confocal scanning laser microscopy (Leica SP5) with a 40× oil immersion objective (NA = 1.25). Retinae were mounted on glass slides under a cover slip in Prolong Gold. Optical sections were collected every 250 nm, with 8-bit depth and a pixel size of 160 nm×160 nm. Each channel was scanned separately and images were line averaged. Retinae are approximately 100 microns deep with a maximal diameter of approximately 500 µm. Individual ommatidia radii are in the range 3–5 µm, and the distance between neighboring ommatidial centers is 12–15 µm. The centers of PRs within an ommatidium are approximately 2 µm apart, while the spacing between each PR is less than 250 nm, and thus just within the resolution limit. A single image stack typically consisted of ∼300 optical slices and saved as 3–4 GB of data (Figure 2A).

### Image Analysis Algorithm

#### Step 1: Identifying ommatidia in an optical slice

To identify ommatidia, the following algorithm is used (Figure 3A). First, in each section, a maximum value filter is applied in a sliding window of size 5 pixels by 5 pixels, chosen because a typical ommatidial cross section is composed of 500–1000 pixels and the distance between ommatidia is between 20–40 pixels. In a window, a maximum intensity value is calculated and assigned to all pixels in that window. The effect of the filter is to blur differences between PR cells, and emphasize differences between ommatidia and the background. On the resulting image, a contour map of intensity levels is generated, using 20 equally-spaced intensity levels from 0 to 255, using the MATLAB function contourc. Starting at the highest intensity contour, the image is thresholded, connected components are identified and the number of pixels that make up the regions are compared to the standard acceptable range for ommatidia (500–1000 pixels). Continuing to the next contour level, the process is repeated, new regions are identified, and previously identified regions grow in size. Once a region's size falls within the acceptable range, it stops growing. This local thresholding accounts for intensity differences across the retina, and allows both dim and bright ommatidia to be easily identified. The output from this analysis consists, in each slice, of a set of putative ommatidial regions specified as a binary mask (i.e. 1 at each pixel that belongs to a region, 0 otherwise).

#### Step 2: Identifying ommatidia in 3D

To align ommatidial regions between slices, we apply a sliding window operation through the stack (Figure 3B). For every 5 slices, starting at the first slice (the deepest slice in the tissue), we record the positions of pixels of value 1 in each slice, and identify strongly connected components of these pixels in three-dimensions. The resulting regions are called nascent ommatidia. Sliding the window of 5 slices up by 1 slice, the procedure is repeated, and regions are identified. Regions which overlap a nascent ommatidium are added to that ommatidium. Regions which fall within an acceptable size range but do not overlap with any regions in previous slices are added as new nascent ommatidia. The procedure is continued up the stack.

We apply simple corrections that catch most errors that may occur in this process. Due to intensity differences across the retina, occasionally the contouring procedure in Step 1 fuses two ommatidia into a single region. To correct for this, every 20 slices, we calculate the average size of an ommatidium's assigned regions. If a new region is greater than one standard deviation from this average, only the portion that directly overlaps with the nascent ommatidium is assigned. The remaining portion is tested for overlap with adjacent ommatidia. If it overlaps, it is re-assigned to a different ommatidium; otherwise it becomes a new nascent ommatidium.

The approach ensures that anomalies within a slice are not erroneously identified as ommatidia, and that differences in ommatidial orientations throughout the retina do not disrupt the three-dimensional alignment of ommatidial regions. The method also corrects for any residual errors due to thresholding. For example, if regions of a slice have been thresholded at a high level resulting in abnormally small regions – i.e. one ommatidium may be broken into two small regions – these small regions will both overlap with one region in subsequent slices and will be joined as one ommatidium. A representative segmented retina is shown in Figure 3C.

#### Step 3: Tracing PR cells within ommatidia

Within ommatidia, the inter-PR distances are at the limits of the resolution of the microscope (<0.5 µm). Ommatidia whose major axis is perpendicular to optical sections are closest to the center of the retina. These ommatidia are imaged at highest resolution and their PRs are easily resolved. Single PRs are more difficult to resolve within ommatidia that are further from the center of the retina, since the resolution in z is lower than resolution in x-y. Taking advantage of the cylindrical geometry of cells, we apply a clustering method on the Euclidean distances between points to identify 7 cells (Figure 3D). Beginning at the R7 layer where cells are furthest apart, we apply k-means clustering [40], where k = 7, to the 800 most intense points in the first five slices, randomly seeding the centers. Sliding up one slice in the stack, we use the group centers identified in the first five slices to seed K-means clustering of the 800 most intense points selected from the next five slices, and so on, tracing the seven PRs through each ommatidium. The center cells, R7 and R8, are identified as one cell using these methods. Since our current study focuses on the outer PRs, we do not identify the boundary between R7 and R8 cells, but refer to them as R7/R8.

As cells extend towards the brain, they twist around the center of the ommatidia and shrink in size, becoming difficult to distinguish, due to insufficient resolution. Clustering in this regime is therefore unreliable. In each retina the parts of the image stack at which clustering is unreliable differs, due to differences in the orientation of the sample on the slide and variation in tissue depth. We used the maximal displacement of cell centers between slices as a measure of the goodness of clustering (see Supplementary Methods and Figure S1). Our unsupervised clustering method allows for automatic cell identification with no user input. As further validation, we visually inspected the automatic cell identification in a subset of ommatidia and from this estimate an error rate in identification of <3%.

#### Step 4: Labeling photoreceptor types

To label the PR types within each ommatidium, the following automatic procedure is used. First, we generate a matrix of distances between cell centers (averaged across the optimum z-range). Using the central cells (R7/R8) as a reference we assign cells as R1–R6: The cell furthest from the center cell is labeled R3. Its two nearest neighboring cells are assigned as R2 or R4. The remaining neighbors of both of these cells are labeled as R1 or R5. Of these cells, the cell with no neighbor is assigned as R1. The other cell is assigned R5. Its neighbor closest to R3 is R4 and the other is R6. The cell between R1 and R3 is labeled as R2.

### Quantification of Rhodopsin Levels

#### Effect of tissue heterogeneity on quantification

Due to the inhomogeneous structure of the retinal tissue, different parts of the retina have slight variations of refractive index, which result in light scattering that can lead to quantification artifacts. For example, when Rhodopsin levels are sufficiently high in one cell, e.g. when Rh6 is expressed at high levels in the R8 cell, some light can be scattered into neighboring cells. Using the contour plot of fluorescence intensity, such behavior manifests as low-intensity contours that emanate from a bright cell and cross into neighboring cells. Our quantification detects and removes this artifact by assigning each intensity contour to a cell if and only if it overlapped with that cell alone, yielding a set of localized contours for each PR (see Figure 4). Contours that overlapped with multiple cells were not used for quantification. Contours that enclosed all 7 cells were collected as a set of contours that characterize the ommatidium's background level (details below).

#### Contour assignment

For each retina, we chose an interval of slices over which all quantification was performed; this set of slices, denoted Z, was semi-automatically identified by our algorithm, as described in Text S1. For a given Rhodopsin channel, within each slice 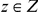, we constructed contour maps of intensity for each ommatidium (see Figure 4). Each intensity contour was assigned to one of several mutually exclusive sets: A_x_ = the set of contours that exclusively overlap PR *x*; B = the set of contours that encompass all PRs. Contour assignment is performed in each *z* slice. First, we identify cell centers as the centroid of all pixels that were assigned to each PR during clustering (see C.3 above). For each cell, we found the minimal distance *d* between its center and the other 6 centers, and placed a circular mask of radius 0.45*d* at the cell's center. Contours that exclusively overlapped the mask associated with PR *x* were assigned to the set A_x_. Contours that enclosed all 7 PR masks were assigned to set B above. For any PRs *x* that were not assigned any contours, we allowed PR masks to increase in radius by no more than 3 pixels, and assigned any contours that overlap exclusively with this larger mask to A_x_.

#### Effect of retinal curvature on quantification

The curvature of the retina results in significant intensity variation across optical sections. The effect is due to the fact that imaging at a given point requires light to travel through the layers of tissue above the point and back to the objective. The retinal curvature is such that at a given z depth, light must pass through more tissue at points further from the center of the retina than at points closer to the center. Figure 4 shows this effect for the Phalloidin fluorescent marker: in a single optical section, the fluorescence intensity is highest for positions in the xy plane that are nearest the top of the retina. For this reason, proper quantification of relative Rhodopsin levels cannot simply be based on the absolute intensity levels across a slice. We therefore compute a local reference intensity level for each ommatidium (details below).

#### Contour averaging and normalization

In each optical section, for each ommatidium we obtain its background contours, and average their intensity values weighted by contour length to obtain the ommatidial reference level, 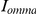. We denote the length of the contour *j* by 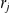, and its intensity level by 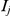. The ommatidial reference level was defined as 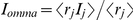, where the averaging is over all 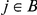. Similarly, for each PR, we obtain its localized contours, and average their intensity weighted by contour length. This yields the average intensity per pixel for the PR, defined as 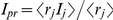, where the averaging is over all 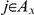. We then compute the *local* relative intensity level of the PR, 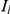, by averaging the ratio 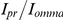 over the set of z slices that are used for quantification: 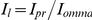.

## Supporting Information

Figure S1 **Optimum z-range landscape.** We plot the landscape that is generated for each retina as described in Supplementary Methods. At each interval length, we plot the number of ommatidia (given by the number on each line) with a particular maximum cell displacement, here defined as the average displacement of a cell's center between two consecutive slices.
(EPS)

Figure S2 **Rhodopsin distributions of replicates using** ***local*** **relative intensity.** Distributions of local relative intensity levels (*I_l_*) are shown for different Rhodopsins at different developmental time points. Histograms are normalized to one, so that the values of the y-axis represent the proportion of PR cells. Each colored line corresponds to a different replicate at the given time point. Numbers of replicates for each panel is given in Figure 6.
(EPS)

Figure S3 **Rhodopsin distributions fit to a single gamma distribution at each time point.**
(EPS)

Figure S4 **Pairwise Rhodopsin correlations.** (I) Data pooled over all replicates. We report the Spearman's ρ correlation coefficient. Significance is indicated as follows: * = mildly significant (0.001<p-value<0.01), ** = significant (0.0001<p-value< = 0.001), *** = strongly significant (p-value< = 0.0001). Left panels: correlation in data pooled across all photoreceptors. Right panels: correlations in data from individual photoreceptor types. (II) Data from individual replicates. Each point indicates the correlation observed in a single replicate. Bars indicate the mean value of the replicates' correlation. Significance of each replicate is shown via color key.
(EPS)

Figure S5 **Stability analysis for the toy network model of Rhodopsin production.** Ribosome production rate is indicated by the blue curve, and degradation rate by the green line. Dotted lines indicate the production threshold *P* ^*^.
(EPS)

Table S1 **Results of Kolmogorov-Smirnov tests for PR-specific repression or de-repression.** We report the significance (−log_10_ p-value) of the one-sided Kolmogorov-Smirnov to test for PR-specific patterns of repression or de-repression. We perform three types of comparisons: (1) We compare the distribution of Rhodopsin level of each PR-type against the pooled distribution of the other PRs. (2) We compare the pooled distribution of Rhodopsin levels between a group of PRs consisting of R2, R4 & R5 with one consisting of R1, R3 & R6. (3) We compare the pooled distribution of Rhodopsin levels between co-recruited pairs of PRs (R3 & R4 vs. R2 & R5 vs. R1 & R6).
(PDF)

Text S1 **Goodness-of-Clustering for ommatidia and toy network model for Rhodopsin production.** Describes methods for picking best-clustered ommatidia as well as the volume over which Rhodopsin levels (*I_l_*) are quantified for further analysis. Additionally, includes a description of the toy model describing a minimal network of Rhodopsin production that is sufficient to explain the two-state phenomenological model used in the main text.
(DOC)
