# Supplementary material for: Stochastic De-repression of Rhodopsins in Single Photoreceptors of the Fly Retina
Source: PLoS Comput Biol. 2012 Feb 2;8(2):e1002357. doi: 10.1371/journal.pcbi.1002357 (PMC3271025; doi:10.1371/journal.pcbi.1002357)
Supplement: Table S1 — Results of Kolmogorov-Smirnov tests for PR-specific repression or de-repression. We report the significance (−log10 p-value) of the one-sided Kolmogorov-Smirnov to test for PR-specific patterns of repression or de-repression. We perform three types of comparisons: (1) We compare the distribution of Rhodopsin level of each PR-type against the pooled distribution of the other PRs. (2) We compare the pooled distribution of Rhodopsin levels between a group of PRs consisting of R2, R4 & R5 with one consisting of R1, R3 & R6. (3) We compare the pooled distribution of Rhodopsin levels between co-recruited pairs of PRs (R3 & R4 vs. R2 & R5 vs. R1 & R6). (PDF) [file pcbi.1002357.s006.pdf]

Table S1. Results of Kolmogorov-Smirnov Tests for PR-specific Repression or De-repression

		Rh3				Rh5				Rh6				Phal			
PR comparisons		0w	1w	2w	4w	0w	1w	2w	4w	0w	1w	2w	4w	0w	1w	2w	4w
One-sided (for repression)	PR1 vs Others	1	0.0	0.1	0.0	0.0	0.3	0.0	0.1	0.1	0.0	0.0	0.0	0.9	0.5	0.4	0.3
	PR2 vs Others	1.2	0.0	0.1	1.0	0.2	0.1	0.7	0.1	6.1	5.0	13.5	5.3	2.8	0.2	6.0	0.1
	PR3 vs Others	74.9	15.8	38.9	32.1	1.9	1.1	17.5	26.8	0.3	0.4	0.1	0.4	0.5	1.4	0.3	4.7
	PR4 vs Others	8.2	3.8	5.8	2.9	1.3	0.5	0.4	0.0	8.8	6.9	29.0	23.9	0.2	0.3	0.0	1.2
	PR5 vs Others	0.8	1.8	0.1	0.1	0.1	0.3	0.3	0.0	12.7	3.5	38.4	7.2	3.2	1.6	3.3	0.0
	PR6 vs Others	0.0	0.0	0.0	0.0	1.8	0.0	0.0	0.2	0.1	0.0	0.1	0.1	1.2	0.2	0.0	0.5
One-sided (for de-repression)	PR1 vs Others	20.2	5.7	2.6	22.6	6.8	0.6	2.5	0.2	16.5	5.4	16.4	19.8	0.8	0.8	1.3	0.7
	PR2 vs Others	5.0	4.6	7.9	0.0	0.3	0.4	2.3	2.0	0.0	0.0	0.2	0.0	0.0	0.3	0.0	1.0
	PR3 vs Others	0.0	0.0	0.0	0.0	0.0	0.0	0.0	0.0	6.9	3.0	43.5	8.0	2.9	0.0	2.3	0.0
	PR4 vs Others	0.5	0.2	0.5	0.1	0.1	0.1	0.4	7.3	0.0	0.0	0.0	0.0	2.6	0.5	1.2	0.1
	PR5 vs Others	0.2	0.0	1.1	0.6	0.5	0.4	0.2	1.1	0.4	0.3	0.0	0.8	0.6	0.4	0.0	3.0
	PR6 vs Others	14.7	3.7	5.8	7.6	0.1	0.8	7.7	0.7	2.6	4.0	19.2	12.1	0.9	0.3	4.3	2.7
One-sided (for repression)	PRs 2,4,5 vs PRs 1,3,6	5.9	0.9	0.3	3.6	1.2	0.1	1.3	0.0	32.1	16.7	118.5	47.9	3.0	0.1	5.7	0.3
One-sided (for repression)	PRs 3,4 vs PRs 2,5	28.7	7.1	21.4	9.6	1.1	0.8	5.6	5.1	0.3	0.7	0.1	0.9	0.2	0.8	0.1	5.2
	PRs 2,5 vs PRs 1, 6	10.9	2.1	0.4	13.6	1.7	0.4	4.9	0.0	19.8	11.2	66.6	31.0	3.8	0.9	8.7	0.5
