# Supplementary material for: Stochastic De-repression of Rhodopsins in Single Photoreceptors of the Fly Retina
Source: PLoS Comput Biol. 2012 Feb 2;8(2):e1002357. doi: 10.1371/journal.pcbi.1002357 (PMC3271025; doi:10.1371/journal.pcbi.1002357)
Supplement: Text S1 — Goodness-of-Clustering for ommatidia and toy network model for Rhodopsin production. Describes methods for picking best-clustered ommatidia as well as the volume over which Rhodopsin levels (Il) are quantified for further analysis. Additionally, includes a description of the toy model describing a minimal network of Rhodopsin production that is sufficient to explain the two-state phenomenological model used in the main text. (DOC) [file pcbi.1002357.s007.doc]

# Text S1

## **Goodness-of-Clustering For Ommatidia**

To determine whether an ommatidium is sufficiently well-clustered, we calculate the displacement of the position of the center of each of its cells between two adjacent slices. For each ommatidium, for each cell, we track this displacement over all the z-slices that compose an ommatidium. We compare this displacement profile among ommatidia to identify the z-range, here called an interval length, across which clustering is most reliable. To this end, across a given interval length and over all seven PR cells, we calculate the maximum cell displacement for each ommatidium. At each interval length, we plot the number of ommatidia with a particular maximum cell displacement (Figure S1). The interval length plotted always begins at the minimum possible z position (z_start_). From this landscape, we pick the optimal z-range such that the number of ommatidia and the interval length (z_end_ - z_start_) is maximized and the displacement of cells among those ommatidia is minimized. Our final choice of z-range is such that there are at minimum 100 ommatidia for which the maximum cell displacement between slices is no more than 4 pixels. The z-range can be no less than 20 slices in length. Additionally, we limit our analysis to regions below the z-value at which cells that act as structural support of the ommatidia (pigment cells) appear. Beyond this, cells are no longer perpendicular to the optical section and the majority of information is collected in the lower resolution z sections.

## **Toy Network Model for Rhodopsin Production**

Since photoreceptors have the capacity to produce very large quantities of Rhodopsin, the protein production machinery itself must switch into “high gear” to produce Rhodopsins in large quantities. We construct a minimal network model that is sufficient to explain the two-state phenomenological model used in the main text. Namely,

1. the network is bistable, with high and low production states.
2. switching from low to high states can occur through fluctuations in Rhodopsin levels.
3. once activated, the high state persists even as Rhodopsin mRNA levels drop below the levels necessary for activation.

Because the molecular details (rate constants, interactions, etc) have not been established, this network model is merely one possibility, i.e. a construction, meant to provide intuition and guide further research. After presenting the network, we discuss its relation to Dve buffering.

Since conditions (1)-(3) above are well-established in other bistable systems, such as the *lac* operon [1,2], we construct our minimal network along similar lines. We will consider the system consisting of the Rhodopsin protein (*R*) and Rhodopsin mRNA (*M*). In analogy with the *lac* operon, we let Rhodopsin protein *R* play the role of the inducer of the Rhodopsin production machinery. In reality, Rhodopsin production is a complex process that involves synthesis of a prosthetic group as well as vesicular trafficking to the rhabdomere. In our model, we do not explicitly include the separate steps and processes of induction, since these are not well characterized. We let *f*(*R*) be the production rate of Rhodopsin per transcript, which in analogy with *lac* will be assumed to have a sigmoidal dependence that results from molecular details of the induction pathway. We let *R*^*^ denote the mid-point of the sigmoidal curve, such that for *R* << *R*^*^, production rate $f\left(R\right)$ is low, and *R* >> *R*^*^, $f\left(R\right)$ is high. By a similar induction pathway, Rhodopsin degradation rate $\gamma\left(R\right)$ can be assumed to increase significantly for *R* above the threshold *R*^*^, such that $\gamma\left(R\right)\approx\gamma_{low}$ for *R* << *R*^*^, and $\gamma\left(R\right)\approx\gamma_{high}$ for *R* >> *R*^*^. With these minimal assumptions, we obtain the differential equation for *R*:

$$\frac{dR}{dt}=f(R)M-\gamma(R)R$$

The constants for Rhodopsin production ($k_{p}$) and degradation ($\gamma_{p}$) used in the main text are related to the network model as follows: $k_{p}=f(R)$ and $\gamma_{p}=\gamma\left(R\right)$. As we will see, the system selects for either high or low values of *R*, which therefore gives rise to either high or low values of $k_{p}$ and $\gamma_{p}$.

We analyze the equilibrium stability of the model by examining the intersection points of the curve $f\left(R\right)$ with the curve $\gamma(R)R/M$. Each intersection point is a fixed point of the dynamics. A pictorial analysis is presented in Figure S5, where for simplicity of drawing we have depicted the case where $\gamma(R)=\gamma$is a constant. Allowing the degradation rate to change from $\gamma_{low}$ to $\gamma_{high}$ at the threshold *R*^*^ does not qualitatively change the possible behaviors we now discuss.

Figure S5 shows plots of $f\left(R\right)$ and the line $cR$, with $c=\gamma/M$, for different possible values of the slope *c*. The slope is inversely proportional to *M*. We see that for high and low values of *M*, a single fixed point exists, which is stable (intersection points are stable when the blue curve is above the green to the left of the point, and below the green to the right of the point). For intermediate values of *M*, three fixed points exist, and the two stable ones are indicated in red. This intermediate range corresponds to the bistable regime of the dynamics, and is completely analogous to the behavior of the *lac* operon [1,2].

While the parameter regimes that correspond to *wild-type* and *dve* are not known, we see that this simple network is capable of explaining the existence of two stable states, with fluctuations that can drive the system from one to the other. If, for example, mRNA levels *M* are such that the slope falls in the bistable regime, then a fluctuation that increases Rhodopsin protein levels can drive the system into the high production state. The fluctuation does not need to involve a change in mRNA levels; the way this network is constructed, fluctuation in Rhodopsin protein levels is sufficient to drive the system into the high state. Once there, the system will produce Rhodopsin at high rates, and the Rhodopsin degradation rate will increase according to $\gamma\left(R\right)$. The system will remain stably in the high state, provided *M* remains in the bistable range.

In this network, the role of Dve would be to suppress the bistable regime, and thus provide a buffer against fluctuations. In the presence of Dve, mRNA levels would be sufficiently low that the bistable regime is avoided, and the system has a unique fixed point corresponding to low production. With the removal of Dve, mRNA levels would increase into the bistable regime. Cells would remain stably in the low production state, until a rare fluctuation of Rhodopsin levels would drive the cell into the high state where it would remain stably. Thus, we see explicitly why Dve’s role in this network would be as a buffer: it controls the stability properties of the system, rather than its state. That is, removal of Dve is not sufficient to activate the high production state – the system is stable in the low production state without Dve. Removal of Dve means the system is merely poised to become activated. It takes a fluctuation of Rhodopsin levels to change the state of production.

## References

1. Chung J (1996) On physiological multiplicity and population heterogeneity of biological systems. Chemical engineering science.

2. Vilar JMG, Guet CC, Leibler S (2003) Modeling network dynamics: the lac operon, a case study. J Cell Biol 161: 471–476. doi:10.1083/jcb.200301125
